# Coexistence of Histologically Proven Chronic Lymphocytic Thyroiditis with Other Thyroid Disorders: A Retrospective Study

**DOI:** 10.1055/s-0041-1740626

**Published:** 2022-06-30

**Authors:** G. Gejoe, I.P. Yadev, Amrutha Kumaran, K.S. Swasthik, Meer M. Chisthi

**Affiliations:** 1 General Surgery, Government Medical College, Trivandrum, Kerala, India

**Keywords:** Hashimoto's thyroiditis, papillary thyroid cancer, multinodular goiter, thyroid

## Abstract

**Background** Hashimoto's thyroiditis (HT) is the commonest autoimmune thyroid pathology. It has been reported in increased numbers recently, probably due to the increase in autoimmune diseases across many parts of the world. It is sometimes found associated with other diseases as well as other diseases of the thyroid. There is an unproven association of this condition with thyroid cancer, particularly papillary thyroid carcinoma (PTC).

**Methods** This was a retrospective study performed over a period of 5 years. The objectives of this study were to find out the prevalence of histopathologically proven HT in surgically resected thyroid glands for various indications and its association with other thyroid disorders, especially thyroid malignancies. Total 4,630 patients who underwent thyroidectomy during the study period and met the criteria for inclusion were considered for analysis.

**Results** Histopathologically proven features of HT were present in 1,295 (28%) of the cases. Among these, 445 (34.36%) had only HT while 850 (65.66%) had HT along with other thyroid diseases. The most common disease associated with HT was multinodular goiter (44.2%), followed by PTC (15.2%). Patients with HT exhibited a higher rate of papillary cancer (16.7%) compared with patients without this pathology (13.8%). Statistically significant association between papillary cancer and HT was found among the female patients.

**Conclusion** The prevalence of HT in patients undergoing thyroidectomy is high in the studied population. A statistically significant association exists between papillary thyroid cancer and thyroiditis among female patients. This could form the basis for further research along these lines.

Hashimoto's thyroiditis (HT) was first described by Hakaru Hashimoto, a Japanese surgeon and pathologist in 1912. It is the most common autoimmune thyroid disease and the commonest cause of hypothyroidism. 1 The disease occurs in 0.3 to 1.5 per 1,000 individuals worldwide and is found to be more common in females with gender preponderance of 5 to 20 times. 2 The pathophysiological hallmark of HT is diffuse lymphocyte infiltration of thyroid follicles resulting in glandular destruction, fibrosis, and parenchymal atrophy, subsequently causing thyroid dysfunction and occasional development of goiter. 3 Though 90% of patients with HT have high antithyroid peroxidase and antithyroglobulin antibody titers, histological diagnosis is considered more accurate. 4 Sonographic findings of diffuse HT include decreased echogenicity, heterogenecity, hypervascularity, and presence of hypoechoic micronodules with an echogenic rim. 5

In the past, thyroiditis was considered as an uncommon disease incidentally diagnosed by the presence of lymphocytic infiltration in thyroid follicles on histopathology examination. Recently, increased number of thyroiditis has been reported. This could parallel the steady rise in frequency of other autoimmune disorders, mostly in the West and North of the world as compared with the East and South, probably due to modified environmental triggers. 6 7 8 Thyroiditis has been associated with other autoimmune diseases like Type 1 diabetes mellitus, multiple sclerosis, rheumatoid arthritis, celiac disease, vitiligo, and chronic urticaria. 8 9 10 11 The association of HT with thyroid cancer, in particular papillary thyroid carcinoma (PTC), was first described by Dailey et al in 1955. 12 This report underlined a significantly high prevalence of thyroid cancer in HT compared with the general population. Although several publications did support these findings, 13 14 the investigations by Crile 15 in population-based studies of patients with HT challenged this association. Another similar study done by Holm et al 16 in 829 patients added strong support to Crile's 16 findings. Later, Jankovic et al 4 did a systematic literature review in 2013 and concluded that population-based fine needle aspiration studies did not find a statistically significant correlation between HT and PTC. The objective of this study is to find out the prevalence of histopathologically proven HT among patients who underwent thyroidectomy for various indications in our institution and the association of HT with other thyroid diseases, especially thyroid malignancies.

## Materials and Methods

All patients aged 12 years or above, who underwent thyroidectomy for various indications from 2011 to 2015 in the Department of General Surgery, in Government Medical College, Trivandrum, Kerala, India, were included in this retrospective study. The main indications for thyroidectomy were benign thyroid disease with pressure symptoms or cosmetic purposes; suspicious nodule(s) in the thyroid by clinical examination, imaging, or fine needle aspiration cytology (FNAC); and proven thyroid malignancy. All patients had undergone FNAC before surgery except in thyrotoxicosis. Those patients who underwent isthmusectomy alone for relieving pressure symptoms and thyroidectomy for underlying parathyroid disease were excluded. HT was defined histologically by the presence of diffuse lymphocytic infiltrates, lymphoid follicles with reactive germinal centers, Hurthle cell change of the follicular epithelial cells, parenchymal atrophy, and fibrosis.

From the hospital registry, data were abstracted by the residents who were given adequate training about the data abstraction protocol based on a pretested and standardized data abstraction form. We had ascertained the feasibility and availability of information needed for the data abstraction form by a preliminary review of three randomly selected sample case records. We abstracted relevant data of all thyroidectomy cases from 2011 to 2015. Descriptive statistics are reported in mean and standard deviation or median and interquartile range for continuous variables, and in absolute numbers and percentages for categorical variables. Chi-square test with appropriate correction, if needed, was used to find out the association between variables considered under the objective. All statistical analyses were implemented in R statistical software version 3.2.0. The level of significance was set at a *p* -value of 0.05.

## Results

Total 4,631 patients who underwent thyroidectomy in our department for both benign and malignant diseases of the thyroid during the study period and met the criteria for inclusion were considered for analysis. Among this, histopathology report of one patient could not be traced and hence excluded. Age of the patients while undergoing thyroidectomy ranged from 12 to 82 years and the median age was 42 (34.51) years. Among those, 60 patients were below the age of 18 years. In these 60 patients, the indications included abnormal cytology, pressure effects, and cosmesis also. None of these 60 patients were found to suffer from any familial thyroid disorders. Postoperative histology revealed papillary cancer as the predominant finding in these patients, 22 (36.7%), followed by benign multinodular goiter (MNG), 15 (25%), lymphocytic thyroiditis, 15 (25%), cellular nodule, 4 (6.7%), and papillary cancer in a background of MNG, 4 (6.7%). The preoperative cytology results are displayed in Table 1 .

**Table 1:** Cytology distribution of all study patients

Multinodular goiter (MNG)	70.8%
Lymphocytic thyroiditis	9.8%
Papillary carcinoma	7.4%
Nondiagnostic	5%
Follicular neoplasm	4.6%
MNG with thyroiditis	3.2%
Papillary carcinoma with thyroiditis	0.7%
Hurthle cell neoplasm	0.5%
Normal thyroid cells	0.27%
Medullary carcinoma	0.2%

Most of them were female 4,075 (88%) with a female-to-male ratio of 7:1. The mean age of the patients who presented with thyroiditis was 41.2 ± 11.8 years, whereas in patients without thyroiditis, the age was 43.3 ± 12.2 years. Even though thyroiditis and nonthyroiditis patients showed female predominance, it was more so with thyroiditis patients. Among thyroiditis patients, females constituted 93.3% (female-to-male ratio, 14:1), and in nonthyroiditis patients, females constituted 85.9% (female-to-male ratio, 6:1). Out of the 4,630 thyroidectomies, 508 had undergone hemithyroidectomy, 29 had subtotal thyroidectomy, 87 had near-total thyroidectomy, and 4,006 had total thyroidectomy. So, the most frequent thyroid surgery performed was total thyroidectomy (86.5%).

**Fig. 1:**
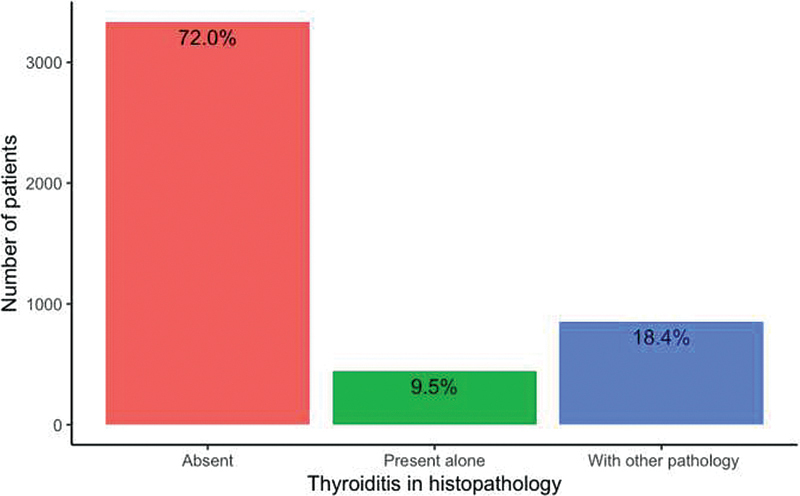
Bar graph showing the distribution of thyroiditis in histopathology.

**Fig. 2:**
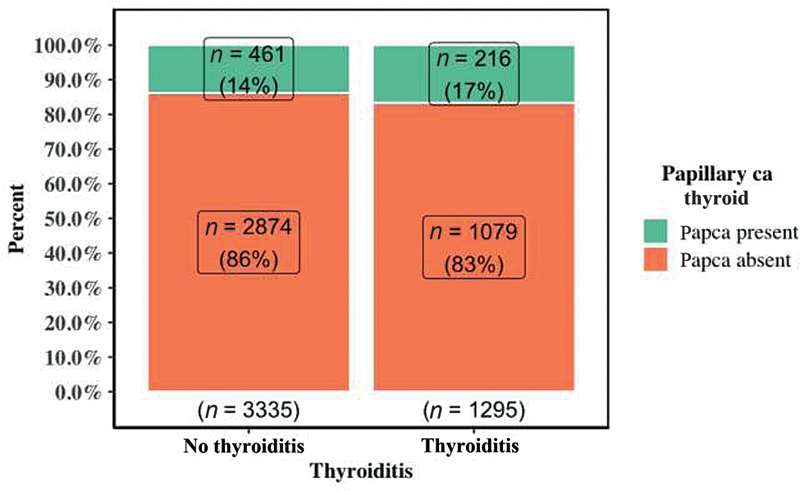
Grouped bar graph showing association between thyroiditis and papillary thyroid cancer.

In this study of 4,630 patients, histopathologically proven features of HT were present in 1,295 (28%) patients, of which 445 (34.36%) had only HT while 850 (65.64%) had HT along with other thyroid diseases. These 445 patients underwent surgery because of pressure effects. The frequency distribution of thyroiditis in thyroidectomy specimens is given in Fig. 1 . The most common disease associated with HT was MNG (44.2%), followed by PTC (15.2%), while 1.5% patients had both MNG and PTC. Other diseases in the decreasing order of frequency were cellular nodule, follicular carcinoma, follicular adenoma, medullary carcinoma, other malignancy, and Hurthle cell carcinoma. Altogether, HT coexists with PTC in 216 (16.7%) cases, with other malignancies in 18 (1.38%) cases, and with other benign thyroid diseases in 616 (47.6%) cases ( Table 2 ). Moreover, patients with HT exhibited a higher rate of PTC compared with patients without HT, irrespective of their gender (16.7% vs 13.8%, *p* = 0.013). Association between thyroiditis and papillary thyroid cancer is depicted in Fig. 2 . Male patients who underwent thyroidectomy harbored PTC more often than females irrespective of their thyroiditis status. When 23% of male HT patients and 20.2% of males without HT suffered from PTC, only 16.2% and 12.8% of females in the HT and without HT group, respectively, had PTC. Statistically significant association exists between PTC and thyroiditis in female patients ( *p* = 0.003) whereas it is lacking in males ( *p* = 0.56). The occurrence of PTC in HT patients and without HT patients with respect to gender is given in Table 3 .

**Table 2:** Hashimoto's thyroiditis' coexistence with other thyroid pathologies

Thyroid disorder	Number (%)
Multinodular goiter (MNG)	573 (44.2)
Hashimoto's thyroiditis only	445 (34.4)
Papillary carcinoma	197 (15.2)
Cellular nodule	32 (2.47)
MNG with papillary carcinoma	19 (1.47)
Follicular carcinoma	11 (0.85)
Follicular adenoma	11 (0.85)
Medullary carcinoma	4 (0.31)
Hodgkin's lymphoma	1 (0.08)
Hurthle cell carcinoma	1 (0.08)
Secondary from unknown primary	1 (0.08)
Total	1,295 (100)

**Table 3:** Papillary cancer co-occurrence in patients with and without Hashimoto's thyroiditis

Sex	Thyroiditis present ( *n* = 1,295)		Thyroiditis absent ( *n* = 3,395)		*p* -Value
	Total no.	PTC (%)	Total no.	PTC (%)	
Female	1,208	196 (16.2)	2,866	366 (12.8)	0.003*
Male	87	20 (23)	469	95 (20.2)	0.56
Total	1,295	216 (16.7)	3,335	461 (13.8)	

Abbreviation: PTC, papillary thyroid carcinoma.

## Discussion

Since the initial description by Dailey et al 12 in 1955, the association between HT and thyroid malignancy remains controversial, some studies suggesting a positive correlation while others strongly contradicting this. Some studies even postulate a cause and effect relationship between the two. The inflammatory response seen in HT stimulates the malignant transformation of follicular cells through reactive oxygen mediated DNA damage. The conflicting report seen in the literature may be due to differences in study design, selection bias, and ethnic and geographical variations. In this study, we attempted to find out the prevalence of HT in thyroidectomy in the Indian population and whether there exists any relationship between HT and PTC.

The prevalence of HT in this study was 28%; of this, 18.4% had HT along with other thyroid diseases and 9.5% had only HT. A study done in Korea by Yoon et al 17 reported a prevalence of 28.7% HT among PTC patients. But similar studies done by Repplinger et al 18 and Siriweera and Ratnatunga 19 in thyroidectomy patients showed prevalence of HT as 18% and 6.51%, respectively, based on final pathology. We focused our study on the distribution of various other thyroid disorders in the subgroup of patients with pathologically proven HT. Among this, 65.64% had associated thyroid pathologies, either benign or malignant. Overall, benign disease of the thyroid was more frequently associated with HT (47.6%) than malignancy (18%) and among the malignancies, PTC formed an overwhelming majority. Since the association between HT and PTC has been widely disputed in the literatures and both these diseases are common in our setting, we further explored the relationship. Out of the 1,295 patients with HT, 16.7% had coexistent PTC and it was 12 times more common than other thyroid malignancy. This could also be because PTC is the commonest type among all thyroid malignancies. If stratified by gender, females were more frequently affected by thyroid cancer than males, with female-to-male ratio of 9.6:1. There is a statistically significant association between PTC and thyroiditis in female patients ( *p* = 0.003) whereas it is lacking in male patients. This may be because of the small sample size of male patients or due to the fact that males with thyroid nodules are often advised thyroidectomy with a lower threshold.

Resende de Paiva et al 20 conducted a large systematic review and meta-analysis involving 64,628 subjects to find out the association between HT and thyroid cancer. Among the HT patients, most of the patients were women (78.7%). PTC was seen in 9.03%, follicular carcinoma in 1.26%, medullary carcinoma in 1.62%, anaplastic carcinoma in 0.49%, and thyroid lymphoma in 0.37%. He concluded that an association exists between HT and PTC as well as HT and thyroid lymphoma, but no association was found between HT and other thyroid malignancies. In all subtypes of thyroid cancer, females were more often affected than males with ratios ranging from 1.5:1 to 4.8:1. These findings go well with our study. Daniel Repplinger et al 18 found that PTC occurred in 29% of patients with HT and 23% of patients without HT. Though PTC was the most common malignancy in patients with or without HT, it was significantly less common in non-HT group (94% vs 76%, *p* = 0.001). A histopathology study assessing the prevalence and severity of thyroiditis among surgically resected thyroid tumors found a significantly higher rate of lymphocytic infiltrate in PTC. 21 Most of the thyroidectomy specimen studies reported a positive correlation between HT and PTC. 3 22 23 24 However, FNAC studies done in the outpatient setting did not find a statistically significant association between the presence of HT and PTC. For example, Matesa-Anić et al 25 analyzed FNAC of 10,508 patients and found that the prevalence of PTC in patients with HT was 1.9% and patients without HT was 2.7%. A similar observation was seen in other FNAC studies. 16 26

To date, the causative relationship between HT and PTC is not clearly established, though there are some proposed mechanisms in the literature. Wirtschafter et al 27 and Arif et al 28 in two different studies demonstrated expression of the RET/PTC1 and RET/PTC3 oncogenes in HT. Further study by Unger et al 29 found the expression of p63 in HT patients with PTC. Burstein et al 30 hypothesized that both HT and PTC are initiated by pluripotent p63-positive stem cell remnants. Another hypothesis states that elevated levels of thyroid stimulating hormone found in HT patients with hypothyroidism stimulate follicular epithelial proliferation, leading to PTC. 15 31 32

Our study was on a large series of patients who underwent thyroidectomy for various indications mentioned above, over a span of 5 years. The limitation of this study is that this is a hospital-based retrospective study comprising thyroidectomy patients alone, hence subjected to selection bias. Also, we could not find complete information about these patients, including their thyroid hormone status and thyroid antibodies status.

But population-based studies in the literature with FNAC for diagnosing HT and coexistent PTC have met with several problems. The presence of HT in a patient can be confirmed only by histopathology examination of the entire gland. Focal HT and small PTC can be missed by FNAC due to sampling error. Furthermore, follicular cell changes associated with HT can be mistaken for thyroid neoplasm. In addition, several studies did not have a control group. A prospective study in a community or outpatient setting with clinical, imaging, antithyroid antibodies, and ultrasound-guided FNAC as a diagnostic tool for HT would probably address this issue. In the future, a prospective histopathology study of thyroid specimens obtained from a large number of subjects is required to establish the association between HT and PTC conclusively.

## Conclusion

The prevalence of HT in patients undergoing thyroidectomy is high in Kerala state in India. Benign diseases of the thyroid are more frequently associated with HT than malignancy. A statistically significant association exists between papillary cancer and thyroiditis in female patients. We recommend that all patients with HT undergo periodic thyroid evaluation to exclude the development of papillary cancer. We also recommend further research to elucidate the association between thyroiditis and thyroid malignancy.
